# Evaluation of the effect of the COVID-19 pandemic on the all-cause, cause-specific mortality, YLL, and life expectancy in the first 2 years in an Iranian population—an ecological study

**DOI:** 10.3389/fpubh.2023.1259202

**Published:** 2023-10-19

**Authors:** Zahra Pirayesh, Seyed Mohammad Riahi, Ali Bidokhti, Toba Kazemi

**Affiliations:** ^1^Student Research Committee, Birjand University of Medical Sciences, Birjand, Iran; ^2^Department of Community Medicine, School of Medicine, Cardiovascular Diseases Research Center, Birjand University of Medical Sciences, Birjand, Iran; ^3^Cardiovascular Diseases Research Center, Birjand University of Medical Sciences, Birjand, Iran; ^4^Department of Cardiology, Cardiovascular Diseases Research Center, Birjand University of Medical Sciences, Birjand, Iran

**Keywords:** COVID-19, mortality, all-cause mortality, years of life lost, life expectancy

## Abstract

**Background:**

COVID-19 pandemic resulted in excess mortality and changed the trends of causes of death worldwide. In this study, we investigate the all-cause and cause-specific deaths during the COVID-19 pandemic (2020–2022) compared to the baseline (2018–2020), considering age groups, gender, place of residence, and place of death in south Khorasan, east of Iran.

**Methods:**

The present ecological study was conducted using South Khorasan Province death certificate data during 2018–2022. The number of death and all-cause and cause-specific mortality rates (per 100,000 people) were calculated and compared based on age groups, place of residence, place of death, and gender before (2018–2020) and during the COVID-19 pandemic (2020–2022). We also calculated total and cause-specific years of life lost (YLL) to death and gender-specific life expectancy at birth.

**Results:**

A total of 7,766 deaths occurred from March 21, 2018, to March 20, 2020 (pre-pandemic) and 9,984 deaths from March 21, 2020, to March 20, 2022 (pandemic). The mean age at death increased by about 2 years during the COVID-19 pandemic. The mortality rate was significantly increased in the age groups 20 years and older. The most excess deaths were recorded in men, Aged more than 60 years, death at home, and the rural population. Mortality due to COVID-19 accounted for nearly 17% of deaths. The highest increase in mortality rate was observed due to endocrine and Cardiovascular diseases. Mortality rates due to the genitourinary system and Certain conditions originating in the perinatal period have decreased during the COVID-19 pandemic. The major causes of death during the pandemic were Cardiovascular diseases, COVID-19, cancer, chronic respiratory diseases, accidents, and endocrine diseases in both sexes, in rural and urban areas. Years of life lost (YLL) increased by nearly 15.0%, which was mostly due to COVID-19, life expectancy at birth has steadily declined from 2018 to202 for both genders (from 78.4 to 75).

**Conclusion:**

In this study, we found that All-cause mortality increased by 25.5% during the COVID-19 pandemic, especially in men, older adult, Rural residents, and those who died at home (outside the hospital). Considering that the most common causes of death during the COVID-19 pandemic are also non-communicable diseases. It is necessary to pay attention to non-communicable diseases even during the pandemic of a serious infectious disease like COVID-19. The years of life lost also increased during the COVID-19 pandemic, which is necessary to pay attention to all age groups, especially the causes of death in young people. In most developing countries, the first cause of death of these groups is accidents.

## Background

Coronavirus disease 2019 (COVID-19) caused by severe acute respiratory syndrome coronavirus 2 (SARS-CoV-2) emerged in Wuhan in December 2019 and rapidly spread across the world (1). The World Health Organization (WHO) declared the COVID-19 pandemic in March 2020 (2). In Iran, on February 19, 2020, two patients were confirmed as SARS-CoV-2 positive in Qom, and the disease rapidly spread all over the country (3).

Wide-spread societal responses to the COVID-19 pandemic dramatically changed human interactions and movements as well as the access to and delivery of healthcare resulting in disruptions in healthcare services that have affected the supply and demand of non-communicable disease (NCD) care, which created the potential for other significant changes to mortality patterns that are not directly attributable to COVID-19 (1, 4). It has been well reported in the studies that patients with NCD (such as cardiovascular diseases, hypertension, diabetes Mellitus, congestive heart failure, chronic kidney disease, and cancer), males, and older adults have an increased risk of death due to COVID-19 (5, 6).

More than 5 million deaths have been reported worldwide due to COVID-19, and more than 144,000 deaths have been reported in Iran from January 2020 to January 2023.^1^ Most studies on COVID-19 mortality have mainly focused on attributable deaths to COVID-19 and the overall excess deaths during the pandemic compared with those in previous years (7, 8). However, the analysis of Causes of Death (CoDs) is necessary to understand all effects of the COVID-19 pandemic on mortality (9).

Studies reported a significant increase in cardiovascular diseases, diabetes, Alzheimer’s disease, and dementia and a decline in mortality due to road traffic accidents and suicide. Also, COVID-19 has negatively affected the outcome of low-energy trauma in older adults, e.g., the 30-day mortality has increased in patients admitted with hip fractures during the pandemic in the UK (1, 9–14).

Studies in Minnesota and São Paulo demonstrated the most excess mortality among older, male, and non-rural residents in 2020 compared with 2018–2019 (before and after the COVID-19 pandemic) (1, 11).

In Iran, most studies in this regard investigated attributable mortality to COVID-19 or the all-cause mortality rate and did not examine cause-specific mortality rates. In a time series study from March 21, 2013, to March 19, 2020, all-cause mortality was investigated, revealing excess mortality during fall and winter, which might be attributed to COVID-19 and the influenza epidemic (15).

In this study, all-cause and cause-specific mortality rates, life expectancy and YLL were compared before and during the COVID-19 pandemic overall and in gender (male and female), place of residence (urban and rural), place of death (home and hospital) and age subgroups in South Khorasan Province, east of Iran, to reveal the effect of COVID-19 pandemic on the all-cause and cause-specific mortality rates, since COVID-19 pandemic is a unique event which provides a valuable experience which can help us in future pandemics.

## Methods

### Study design and data sources

The studied population of this ecological study consisted of all individuals in South Khorasan Province during 2018–2022. Population data were obtained from the Planning and Budget Organization of South Khorasan Province, We included all individuals from March 21, 2018, to March 20, 2022, registered in South Khorasan. Which included the detailed estimated population of South Khorasan Province in gender, place of residence, place of death, and 5-year age groups for the years 2018 to 2022. The Information of the deceased based on death certificate data for four years was divided into two primary periods: 2018–2019 (pre-pandemic) and 2020–2022 (pandemic). Decedent demographics taken from the death certificate, including age, sex, place of death, place of residence, and underlying cause of death (based on the ICD10 code), were obtained from the Death Registration System of South Khorasan Health Department. The all-cause and cause-specific mortality rates per 100,000 population based on age and gender and ICD-10 chapters were calculated before and during the COVID-19 pandemic. All-cause and cause-specific mortality rates were compared between the two primary periods of pre-pandemic (2018–2019) and pandemic (2020–2022). The all-cause mortality rate was also compared in gender (male/female), place of residence (urban/rural), place of death (home/hospital), and, age (0–19, 20–29, 30–59, 60, and over) subgroups (11), and relative risk between subgroups were calculated. To calculate the denominator of the mortality index fraction in two periods before and after of COVID-19 in this study, the middle population of the South Khorasan Province in the 2-year periods studied was used.

### Statistical analysis

Total and cause-specific years of life lost (YLL) to death were also calculated before and during the COVID-19 pandemic by multiplying the number of deaths in each age and gender subgroup by the residual life expectancy in Iran provided by the World Health Organization (16). Overall and gender-specific life expectancy at birth was also calculated using Analyzing Mortality and Cause of Death (version 3) (ANACod3), which is an online tool developed by the World Health Organization (WHO) for analysis of causes of death that provides several indicators, including life expectancy at birth (17). For each cause of death, demographic-specific rates of death were compared between 2018–2019 and 2020–2022. *p*-values below 0.05 were considered statistically significant, and 95% confidence intervals (95% CI) were calculated. All statistical analyses were performed using Microsoft Excel software and IBM SPSS (version 26).

## Results

A total of 17,750 deaths were recorded in the study timeframe in South Khorasan Province, of which 7,766 deaths occurred from March 21, 2018, to March 20, 2020 (pre-pandemic) and 9,984 deaths from March 21, 2020, to March 20, 2022 (pandemic). The mortality rate per 100,000 population increased from 976.86 before the COVID-19 pandemic to 1226.54 during the COVID-19 pandemic. During the COVID-19 pandemic, the mean age of death increased by about 2 years (from 66.78 ± 26.4 years to 68.59 ± 24.3 years), which shows that the most excess deaths occurred in the age group 60 years and over.

The most remarkable excess deaths were recorded in the age group of 60 years and over [OR = 1.30, 95% CI 1.30 (1.25–1.35), *p* < 0.001], male gender [OR = 1.28, 95% CI (1.23–1. 34), *p* < 0.001] and rural population [OR = 1.29, 95% CI (1.24–1.38), *p* < 0.001]. The death that happened at home increased more than death in hospital [OR = 1.33, 95% CI (1.27–1. 40), *p* < 0.001] (Table 1).

**Table 1:** South Khorasan all-cause mortality rate per 100,000 population, by demographic subgroup, 2018–2022.

	Pre pandemic (*N* = per 100,000)	Pandemic (*N* = per 100,000)	*p*-value	RR* (95% CI)
Age group (years)				
0–19	742 (254.98)	705 (238.58)	0.194	0.93 (0.84–1.03)
20–29	206 (154.89)	241 (186.82)	0.048**	1.20 (1.00–1.45)
30–59	1,147 (390.80)	1,526 (494.65)	<0.001**	1.26 (1.17–1.36)
60 and over	5,671 (7317.42)	7,512 (9274.07)	<0.001**	1.27 (1.23–1.31)
Place of residence				
Urban	4,000 (831.60)	5,155 (1024.85)	<0.001**	1.23 (1.18–1.28)
Rural	3,766 (1197.46)	4,829 (1550.24)	<0.001**	1.29 (1.24–1.38)
Gender				
Male	4,259 (1036.99)	5,535 (1330.53)	<0.001**	1.28 (1.23–1. 33)
Female	3,560 (908.16)	4,448 (1113.39)	<0.001**	1.22 (1.17–1.27)
Place of death				
Home	3,089 (387.8)	4,217 (518.1)	<0.001**	1.33 (1.27–1.39)
Hospital	3,722 (467.3)	4,781 (587.3)	<0.001**	1.25 (1.20–1.31)

*Reference group for calculation RR is Pre pandemic category. **: statistically significant.

**Figure 1:**
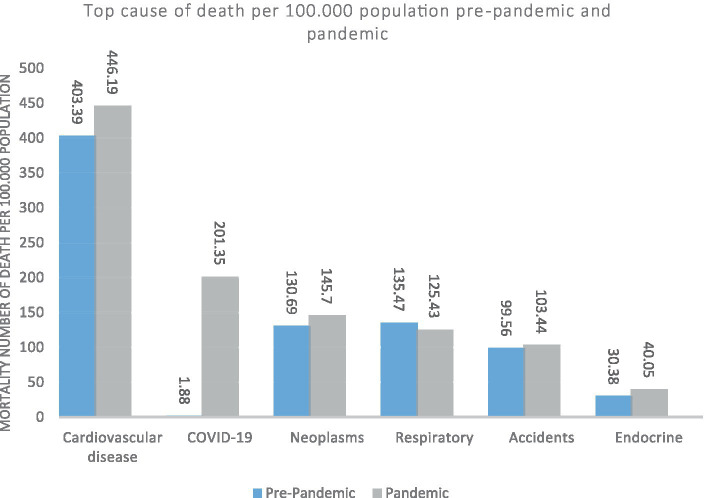
South Khorasan Province, the top causes of death (mortality rate per 100,000 population) before and after of COVID-19 pandemic.

Figure 1 shows the most common causes of death before and during the COVID-19 pandemic. Before COVID-19, the most common causes of death were Cardiovascular, respiratory, neoplasm, and accidents. During the COVID-19 pandemic, the most common causes of death were Cardiovascular, COVID-19, neoplasm, respiratory, and accidents.

**Figure 2:**
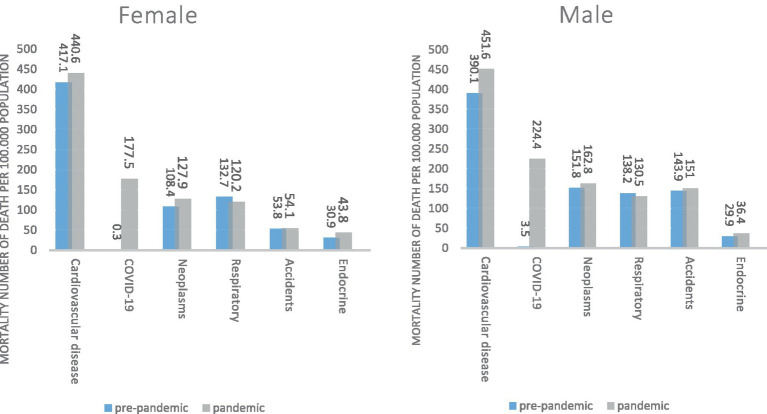
South Khorasan Province, the top causes of death (mortality rate per 100,000 population) based on gender pre-pandemic and pandemic.

Figure 2 shows the most common causes of death before and during the Covid-19 pandemic by sex. In both genders, the most common causes of death before and after COVID-19 pandemic, Cardiovascular diseases, and during the pandemic, COVID-19 is the second cause of death in both sexes. Death due to cardiovascular diseases, neoplasms, and endocrine diseases has increased in both sexes during the COVID-19 pandemic. Death due to respiratory diseases during the COVID-19 pandemic has decreased in both sexes, death due to accidents has increased in men and decreased in women.

**Figure 3:**
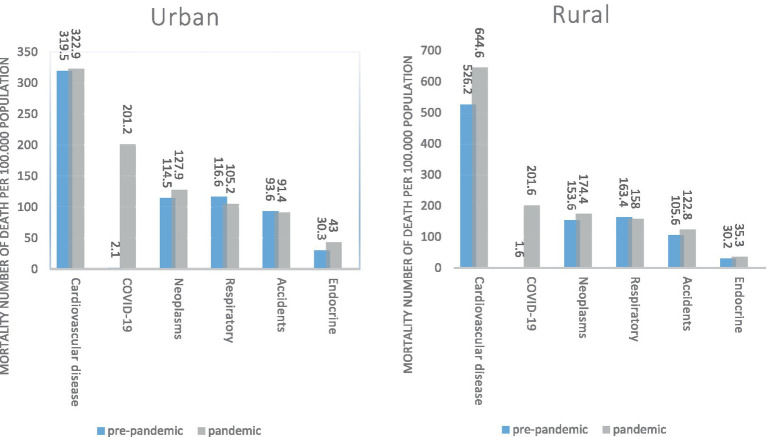
South Khorasan Province, the top causes of death (mortality rate per 100,000 population) based on residence status pre-pandemic and pandemic.

Figure 3 shows the most common causes of death before and during the COVID-19 pandemic based on the place of residence. In urban and rural areas, the most common causes of death before and after COVID-19 pandemic cardiovascular diseases, and during the pandemic, COVID-19 is the second leading cause of death in both locations. Mortality due to cardiovascular diseases (especially in the rural area), neoplasms, and endocrine diseases have increased in both the rural and urban during the COVID-19 pandemic. Deaths due to respiratory diseases have decreased in both places during the COVID-19 pandemic, but deaths due to accidents have increased in the rural and decreased in the urban area.

**Figure 4:**
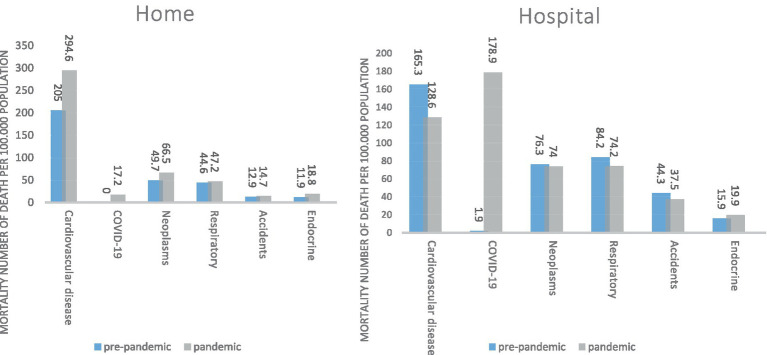
South Khorasan Province, the top causes of death (mortality rate per 100,000 population) based on place of death pre-pandemic and pandemic.

Figure 4 shows the causes of death according to the place of death before and during the COVID-19.

It is noteworthy that the highest mortality rate of COVID-19 was in the hospital than at home.

On the other hand, death due to Cardiovascular diseases was significantly higher during the COVID-19 pandemic at home.

**Figure 5:**
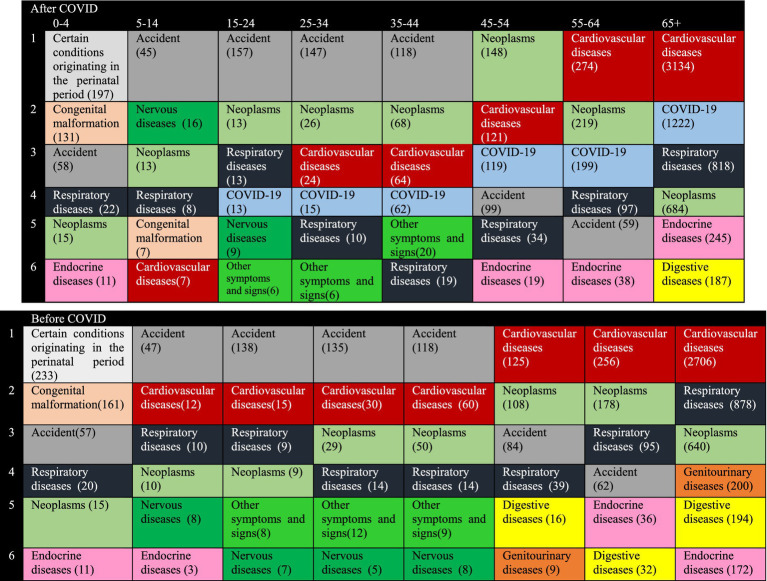
South Khorasan Province, the top causes of death (mortality rate per 100,000 population) based on age group pre-pandemic and pandemic.

Figure 5 shows the most common causes of death according to different age groups before and during the COVID-19 pandemic. The important and debatable points are that, Death due to cardiovascular diseases is the first cause of death in both periods in people over 45 years old. Also, from the age of 5 to 44 years, accidents are the most common cause of death in both periods.

In Table 2, Causes of death and its changes during this period have been shown. As can be seen, during the COVID-19 pandemic, death due to endocrine diseases, cardiovascular diseases, and death with unknown causes has increased statistically, and death due to genitourinary diseases and perinatal period has decreased. Other causes did not increase or decrease significantly.

**Table 2:** South Khorasan Province, the cause-specific mortality rate per 100,000 population before and after of COVID-19 pandemic.

Year	Pre pandemic (*N* = per 100,000)	Pandemic (*N* = per 100,000)	OR (95% CI)	*p*-value
The population of South Khorasan	796,500	814,000		
Infectious and parasitic diseases (excluding COVID-19)	99 (12.43)	79 (9.71)	0.78 (0.58–1.04)	0.100
COVID-19	15 (1.88)	1,639 (201.35)	107.13 (64.43–178.11)	<0.001^*^
Neoplasms	1,039 (130.69)	1,186 (145.70)	1.11 (1.02–1.21)	0.09
Diseases of the blood and blood-forming organs and certain disorders involving the immune mechanism	32 (4.02)	41 (5.04)	1.25 (0.79–1.99)	0.337
Endocrine, nutritional, and metabolic diseases	242 (30.38)	326 (40.05)	1.31 (1.11–1.55)	0.001*
Mental and behavioral disorders	35 (4.39)	27 (3.32)	0.75 (0.45–1.24)	0.271
Nervous diseases	168 (21.09)	154 (18.92)	0.89 (0.72–1.11)	0.329
Diseases of the eye and adnexa	1 (0.13)	1 (0.12)	0.97 (0.06–15.64)	0.988
Cardiovascular diseases	3,213 (403.39)	3,632 (446.19)	1.07 (1.05–1.16)	0.001*
Diseases of the respiratory system	1,079 (135/0.47)	1,021 (125.43)	0.92 (0.85–1.00)	0.078
Diseases of the digestive system	262 (32.89)	246 (30.22)	0.91 (0.77–1.09)	0.340
Diseases of the skin and subcutaneous tissue	7 (0.88)	15 (1.84)	2.09 (0.85–5.14)	0.098
Diseases of the musculoskeletal system and connective tissue	10 (1.26)	11 (1.35)	1.07 (0.45–2.53)	0.866
Diseases of the genitourinary system	252 (31.64)	208 (25.55)	0.80 (0.67–0.97)	0.022*
Pregnancy, childbirth, and the puerperium	6 (0.75)	7 (0.86)	1.14 (0.38–3.39)	0.812
Certain conditions originating in the perinatal period	233 (29.25)	197 (24.20)	0.82 (0.68–1.00)	0.050*
Congenital malformations, deformations, and chromosomal abnormalities	166 (20.84)	143 (17.57)	0.84 (0.67–1.05)	0.134
Symptoms, signs, and abnormal clinical and laboratory findings not elsewhere classified	160 (20.09)	204 (25.06)	1.24 (1.01–1.53)	0.036*
Injury, poisoning, and certain other consequences of external causes	11 (1.38)	5 (0.61)	0.44 (0.15–1.28)	0.123
Accident mortality	793 (99.56)	842 (103.44)	1.03 (0.94–1.14)	0.440
Total	7,766 (982.17)	9,984 (1226.54)	1.25 (1.21–1.29)	<0.001*

* indicate that the *p*-value is significant.

Years of life lost (YLL) increased by nearly 15.0% from 10339.0 to 11885.2 years/100,000 population/year after COVID-19 pandemic compared with before the pandemic. The increased YLL was mostly due to COVID-19 (1535.8 years/100,000 population/years), while minimal changes were observed in other causes of death. YLL due to Cardiovascular diseases and Accidents was slightly increased, while a considerable increase was observed in neoplasms (nearly 15.3% from 1309.1 to 1510.0) and remarkable reductions were seen in certain conditions originating in the perinatal period (nearly 17.5% from 1130.3 to 932.4) and Congenital malformation (nearly 16.7% from 800.9 to 667.1).

**Figure 6:**
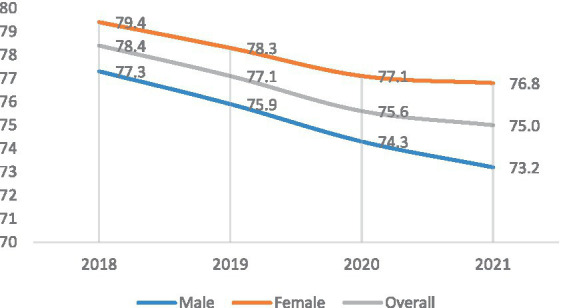
The 4-year trend of life expectancy at birth in South Khorasan Province.

The 4-year trend of life expectancy at birth in South Khorasan Province shows a steadily declining trend for both genders with the most remarkable decline being observed in the first year of COVID-19 pandemic (from 77.1 to 75.6 years). The declining slope of life expectancy was relatively blunted in the second year of the pandemic (from 75.6 to 75.0 years) (Figure 6).

## Discussion

According to our searches, this is the first study in Iran that examines the causes of mortality before and during the COVID-19 pandemic in a region of Iran. Death certificate data in South Khorasan Province showed 25.5% excess deaths in 2020–2022 compared with 2018–2020 (28% for men and 22% for women). which is consistent with the reported results from Latvia (16%), São Paulo (29.6% for men and 20.0% for women), and Minnesota (15.2% for men and 12.8% for women) (1, 4, 11, 18). The urban population had 23% excess in mortality and the rural population had 29% excess during the pandemic, In the other study in Minnesota mortality had excess too (11% for rural and 15% for urban) (1). Mortality occurred in homes over about 33% whereas mortality occurred in hospitals more than 25%. In England, mortality during COVID-19 had excess too (39% for home and 21% for hospital) (19). Men and urban populations were probably at greater risk for COVID-19 than women and rural populations. During COVID-19 many people did not go to the hospital for fear of contracting COVID-19, and the mortality rate increased at home, while the amount of care and follow-up of patients in the hospital increased. The mortality rate was reduced in the 0–19 age group compared with before the COVID-19 pandemic (approximately −7%). Deaths due to Genitourinary diseases (−20%), Respiratory diseases (−8%), and Certain conditions originating in the perinatal period (−17%) had decreased during the COVID-19 pandemic compared with before the COVID-19 pandemic. Moreover, no significant changes were observed in terms of other causes of death, including Nervous diseases and other causes. In terms of gender, more excess deaths due to COVID-19, Cardiovascular disease, and Neoplasms were observed among men; on the other hand, more excess deaths due to Endocrine, nutritional, and metabolic diseases were reported among women. After the COVID-19 pandemic, in terms of place of residence, more excess deaths were reported due to Neoplasms and Endocrine diseases among urban people, while more deaths due to Cardiovascular disease were observed among rural people. Before COVID-19, Cardiovascular diseases were the most important causes of death, after COVID-19 there was a significant increase (10.6%) and they were still at first in South Khorasan Province. In Latvia mortality due to this cause had excess too (4%), while death due to these diseases has significantly reduced in Norway (−19.7%) and São Paulo (−7%), and not significantly changed in Minnesota and South Korea (1, 4, 11, 12, 20). The highest increase in mortality at home was related to Cardiovascular disease which is related to the non-referral of Cardiovascular disease patients to the hospital. The increased rate of mortality due to Cardiovascular disease in South Khorasan and Latvia may be attributed to the challenges of providing healthcare services to patients with cardiovascular diseases and the significant decrease in primary percutaneous coronary intervention procedures during the pandemic (18, 21). A qualitative study by Moslehi et al. showed that limited resources in the cardiovascular department, lack of efficient and effective education, improper hospital preparedness, overcrowding in medical centers, and poor quality and quantity of healthcare services affected the care of patients with cardiovascular diseases (21). Moreover, a generally increasing trend is observed in the number of deaths caused by Cardiovascular disease in Iran and South Khorasan Province, which is mainly due to the gradual population aging, known as health transition (22, 23). On the other hand, a considerable proportion of ill-defined causes of death in Iran belong to Cardiovascular disease; thus, the COVID-19 pandemic and its effect on the quality of causes of death may have affected the excess deaths due to these diseases (18, 24). During COVID-19, cardiac interventions such as PCI have been reduced and the Organization and transport of patients to the hospital have been delayed. Some studies express that COVID-19 may induce acute cardiac injury or myopericarditis, Thromboembolic or atherothrombotic events have increased in the context of this virus (25). Mortality due to COVID-19 accounts for 17% of all causes of mortality and was the second cause of death among both genders, urban and rural populations and the age 60 and over, but it was the third cause of death in the 45–64 age group, In the other age groups, mortality due to COVID-19 was in the lower ranks. Mortality due to COVID-19 was higher in 2020 (the first year of the pandemic) compared with 2022(the second year of the pandemic), which is probably due to vaccination against COVID-19, promotion of public health, and therapeutic experiences. In this study, the average age of those who died due to cardiovascular diseases has increased significantly by 2 years after the COVID-19 epidemic. Excess deaths have been reported in South Khorasan Province due to Neoplasms (11.5%) after the COVID-19 pandemic. Excess deaths due to Neoplasms were reported in Latvia within 3 weeks of their study and had a significant positive excess of death, but the total excess mortality from malignant neoplasms was not significant (4). On the other hand, mortality due to Neoplasms has significantly decreased during the COVID-19 pandemic in South Korea (3%), São Paulo (1.5%), and Minnesota (2%), and did not significantly change in Norway (1, 11, 12, 20). Excess deaths due to Neoplasms in South Khorasan during the COVID-19 pandemic are partly due to the population aging and the health transition (23) as well as the reduced screening and delayed cancer treatments due to limited health care sources available for cancer treatment during the COVID-19 (26). Moreover, the nature of cancer and the immunocompromised state of patients undergoing chemotherapy may have reduced their referral for healthcare services during the pandemic and increased the rate of COVID-19 complications (27, 28). During the COVID-19 pandemic, mortality due to Endocrine, nutritional, and metabolic diseases increased by 33.3% in South Khorasan Province. Mortality related to diabetes mellitus in Norway was also higher than expected (49.9%). In Minnesota, mortality due to diabetes had an excess of approximately 8%. However, mortality due to diabetes mellitus was not significantly changed in São Paulo and South Korea (1, 11, 12, 20). The increasing trend of mortality due to non-communicable diseases, especially diabetes mellitus, has already been reported in epidemiological studies in Iran before the COVID-19 pandemic (29, 30). On the other hand, Nouhjah et al.’s study showed that the self-care behaviors of Iranian diabetic patients using insulin pens had significantly declined during the COVID-19 pandemic (31). Besides, Mirahmadizadeh et al.’s study in Iran showed a significant decrease in visits of diabetic patients by physicians and health workers during the COVID-19 pandemic (32), which might probably affect the mortality due to diabetes. COVID-19 enter cellular access by angiotensin-converting enzyme 2 (ACE2) and transmembrane serine protease 2 (TMPRSS2) which both of them are expressed in many endocrine glands that’s why this virus causes thyroid dysfunction such as thyroiditis, insufficient pancreatic insulin secretion and hyperglycemia or ketoacidosis, adrenal infarction, and disruption of sex hormones in both sexes (33). Mortality due to accidents had an excess of nearly 4%, which was not statistically significant. Consistent with the findings of the present study, The mortality due to accidents increased insignificantly in Norway and Minnesota and decreased by nearly 2% in South Korea (1, 12, 20). Even before and during COVID-19, accidents were the most important cause of death in the 5–44 age group, which shows that COVID-19 has not been able to cause a significant impact on this age group and to reduce the death of young people, the number of accidents should still be reduced. A study in Shiraz, Iran showed reduced hospital admission due to traffic accidents during the COVID-19 pandemic; though, they reported increased mortality due to road traffic accidents which are probably due to reduced traffic safety leading to more lethal accidents (34). Similarly, Shaik and Ahmed investigated the effect of COVID-19 on road traffic crashes. They reported that COVID-19 has reduced traffic flow and increased risky driving behaviors leading to fewer, though more serious, traffic accidents (35). Contrarily, earlier studies, such as Wegman and Katrakazas study, showed reduced vehicle kilometers by 10% and mortality due to COVID-19 by 12.9% in the 24 investigated countries early in the COVID-19 pandemic (36). Mortality due to Symptoms, signs, and abnormal clinical and laboratory findings not elsewhere classified increased by about 24% significantly, This cause of death includes deaths due to unknown causes, this excess may be related to excess death at home that they often do not have an exact cause, and a verbal autopsy is needed for accurate diagnosis. Whereas during the COVID-19, there wasn’t enough time and a Lack of familiarity among general practitioners with verbal autopsy methods. Mortality due to the respiratory system decreased by about 8% % in South Khorasan Province. Also in South Korea mortality related to respiratory diseases decreased by about 12.8% (37); it may be related to the promotion of levels of personal hygiene and mask-wearing from the beginning of the pandemic, which are established major factors in the decrease in respiratory infections. However, in a similar study in Pavia Province Italy, mortality due to respiratory system excessed about 30% among women and 40% among men, and in another one in Rome, mortality due to respiratory system excessed among men only (38, 39). Mortality due to genitourinary diseases in South Khorasan Province decreased by about 20% during the COVID-19 pandemic which was statistically significant. In a similar study in South Korea, mortality due to genitourinary diseases exceeded about 1% in South Korea, and about 23% in England (19, 37). On the other hand, the mortality due to genitourinary diseases did not remarkably change among women while it was reduced by nearly 20% among men in Pavia Province (39). Also in Costa Rica, mortality due to this cause decreased (40). Reducing the incidence of sexually transmitted diseases and reducing the use of nephrotoxic drugs during the pandemic probably decreased Mortality due to genitourinary diseases. Mortality due to certain infectious and parasitic diseases, nervous diseases and diseases of the blood and blood-forming organs, and certain disorders involving the immune mechanism decreased during COVID-19, though the change was not statistically significant. On the other hand, in Pavia Province in Italy, mortality due to nervous diseases decreased by nearly 8% among men and increased by about 14% among women. Mortality due to Dementia and Alzheimer’s increased in Pavia by nearly 24% among men and by nearly 10% among women (39). Moreover, deaths due to dementia and Alzheimer’s disease increased in England. Mortality due to these diseases mostly occurred at home, while it decreased in the hospital (19). These diseases most commonly affect the older adult who are at high risk of COVID-19; such deaths might be recorded as death due to COVID-19. In the other study reducing access to health care as well as the emotional distress and fear of COVID-19 infection were considered to contribute to increased mortality due to these diseases (41). During the peak of COVID-19 death caused by this virus was at the top of the causes of death, but the death caused by other respiratory and infectious diseases decreased, Outside of this peak, death due to chronic non-communicable diseases such as cardiovascular disease, stroke, and diabetes has increased. It may be related to improving the level of personal hygiene and health promotion of society, reducing visits to medical centers controlling non-communicable diseases, and performing routine check-ups of these patients. The four-year trend of life expectancy at birth in South Khorasan Province showed a remarkable decline after the COVID-19 pandemic; comparing 2020–2022 with 2018–2019, life expectancy was reduced by 2.1 years (from 77.1 to 75.0 years) and males were affected more than women. Other studies showed that life expectancy was also reduced in Mexico (ranging between 0.5–4 years in different cities in 2020), Russia (2 years in 2022), the United States (1.67 years in 2020), and Iran (1.4 years in 2020) during the COVID-19 (42–45). The decrease in life expectancy is probably due to the direct impact of COVID-19 and its indirect impact due to the increase in non-communicable diseases and the high population of older adult people with poor health status in South Khorasan Province. The Years of life lost (YLL) in South Khorasan Province increased by nearly 15% after the COVID-19 pandemic which was mostly due to COVID-19 (1535.8 years/100,000 population/year). A similar study in England and Wales showed that YLL increased by 15% during the COVID-19 pandemic and 1350.4 years/100,000 population/year YLL was reported due to COVID-19 (46). Similar YLLs due to COVID-19 were also reported in studies in the United States (1631.2 years/100.000 populations/year), Spain (664.6 years/100,000 populations/year), Italy (656.0 years/100,000 populations/year), Sweden (413.4 years/100,000 populations/year) during the COVID-19 pandemic (45, 47, 48). The excess in YLL is due to the direct and indirect impact of COVID-19. The male population makes up a larger part of YLL compared to women. Wrongly recording the cause of death in the death certificate due to the excess in the total number of deaths, excess in the number of deaths that occurred at home, and the overcrowding of hospitals and other medical centers, the mysterious and unknown effects of the SARS-CoV-2 on the physiology of the body, absence of time interval, absence of certifier signature, incorrect underlying cause-of-death and competing causes of death increased during the COVID-19 pandemic, which was the most important limitation of this study. Due to the fear of contracting the COVID-19 virus, during the pandemic, detailed examinations may not have been done exquisitely to identify the cause of death of the deceased (49).

## Conclusion

In this study, it was observed that the overall mortality rate has increased significantly during the COVID-19 pandemic. This increase was observed in both sexes, rural and urban residents, in all age groups above 20 years. Among the causes of death, the most common causes were Cardiovascular diseases, cancer, chronic Respiratory diseases, accidents, and endocrine diseases. This shows that there are still non-communicable diseases and it makes us plan to reduce these diseases. Even during the pandemic of a serious infectious disease such as COVID-19, we should not ignore these diseases. Death due to Cardiovascular diseases in homes increased significantly. This issue indicates that we should have centers to provide healthy and safe services for chronic patients during the pandemic of serious infectious diseases so that people are not deprived of service due to the fear of infectious diseases. In developing countries, accidents are still the main reason for the death of young people, so government officials should amend the traffic laws, provide safe vehicles to the people, and make the roads safer. Years of life lost (YLL) increased by nearly 15.0%, most of it was directly due to COVID-19, and YLL due to the other cause has minimal change. Excess mortality may be related to reduced access to healthcare services, delayed therapeutic measures, and altered healthcare behaviors. The factors leading to excess deaths should be carefully identified and addressed to prevent further excess deaths in future pandemics.

## Data availability statement

The raw data supporting the conclusions of this article will be made available by the authors, without undue reservation.

## Ethics statement

The studies involving humans were approved by the ethical cod was obtained (IR.BUMS.REC.1400.423). The studies were conducted by the local legislation and institutional requirements. The participants provided their written informed consent to participate in this study.

## Author contributions

ZP: Conceptualization, Writing – original draft, Data curation. SR: Conceptualization, Writing – original draft, Formal analysis. AB: Formal analysis, Data curation, Writing – review & editing. TK: Conceptualization, Writing – original draft.

## Footnotes

^1^https://covid19.who.int/
